# Supplementary material for: The Association between Childhood Exposure to Ambient Air Pollution and Obesity: A Systematic Review and Meta-Analysis
Source: Int J Environ Res Public Health. 2022 Apr 8;19(8):4491. doi: 10.3390/ijerph19084491 (PMC9030539; doi:10.3390/ijerph19084491)
Supplement: Supplementary file 1 [file ijerph-19-04491-s001.zip › ijerph-1640285-supplementary.pdf]

International Journal of
Environmental Research and Public Health

MDPI

The Association between Childhood Exposure to Ambient Air Pollution and Obesity: A Systematic Review and Meta-Analysis

Specific Search Methods

<u>PubMed:</u> 1699 records

Exposure (1 OR 2 OR 3 OR 4 OR 5 OR 6) AND **Outcome** (1 OR 2 OR 3 OR 4 OR 5 OR 6OR 7 OR 8) AND **Object ()**

Exposure:

1.“air pollution”[MeSH Terms] OR “air pollutants”[MeSH Terms] OR “air pollut*”[All Fields] OR “air quality”[All Fields] OR “air environmental pollut*”[All Fields] OR “environmental air pollut*”[All Fields] OR “ambient air pollut*”[All Fields]

2. “particulate matter”[MeSH Terms] OR “particulate matter” [All Fields] OR “ambient air particulates”[All Fields] OR “airborne particulate matter”[All Fields] OR “particulate air pollutants”[All Fields] OR “ambient particulate matter”[All Fields] OR “fine particles”[All Fields] OR “fine particulate matter”[All Fields] OR “PM_{10}”[All Fields] OR “$PM_{2.5}$”[All Fields] OR “PM_1”[All Fields]

3. “nitrogen dioxide”[MeSH Terms] OR “nitrogen oxide”[All Fields] OR “nitrogen dioxide”[All Fields] OR “NO_2”[All Fields]

4. “sulfur dioxide”[MeSH Terms] OR “sulfur oxides”[All Fields] OR “sulfur dioxide”[All Fields] OR “SO_2”[All Fields]

5. “ozone”[MeSH Terms] OR “ozone”[All Fields] OR “Ground Level Ozone”[All Fields] OR “O_3”[All Fields]

6. “carbon monoxide”[MeSH Terms] OR “carbon monoxide” [All Fields]

Outcome:

1.“obesity”[MeSH Terms] OR “obesit*”[All Fields] OR “obese”[All Fields]

2. “overweight”[MeSH Terms] OR “overweight*”[All Fields]

3. “body weight”[MeSH Terms] OR “body weight*”[All Fields]

4. “obesity, abdominal”[MeSH Terms] OR “abdominal obesit*”[All Fields] OR “abdominal overweight” [All Fields] OR “central obesit*”[All Fields] OR “visceral obesit*”[All Fields]

5. “body mass index”[MeSH Terms] OR “body mass index” [All Fields] OR “BMI”[All Fields] OR “Quetelet Index”[All Fields]

6. “waist circumference”[MeSH Terms] OR “waist circumference”[All Fields] OR “WC”[All Fields]

7. “waist-hip ratio”[MeSH Terms] OR “waist-hip ratio”[All Fields] OR “waist to hip ratio”[All Fields] OR “WHR”[All Fields]

8. “fat body”[MeSH Terms] OR “fat body”[All Fields] OR “body fat”[All Fields] OR “adipose tissue”[MeSH Terms] OR “adipose tissue”[All Fields]

Object:

“child” [MeSH Terms] OR “adolescent” [MeSH Terms] OR “child*” [All Fields] OR “adolescen*” [All Fields] OR “juvenile*” [All Fields] OR “youth*” [All Fields] OR “teen*” [All Fields] OR “kid” [All Fields] OR “kids” [All Fields] OR “pubescent*” [All Fields] OR “pubert*” [All Fields] OR “youngster*” [All Fields] OR “minor*” [All Fields] OR “student*” [All Fields] OR “pupil*” [All Fields] OR “preschooler*” [All Fields] OR “schoolchild” [All Fields]

<u>Web of Science:</u> 2229 records

Exposure TS = (1 OR 2 OR 3 OR 4 OR 5 OR 6) AND **Outcome** TS= (1 OR 2 OR 3 OR 4 OR 5 OR 6OR 7 OR 8) AND **Object** TS = ()

Exposure:

1.Topic = (“air pollut*” OR “air quality” OR “air environmental pollut*” OR “environmental air pollut*” OR “ambient air pollut*”)

2. Topic = (“particulate matter” OR “fine particles” OR “ambient air particulates” OR “PM_{10}” OR “$PM_{2.5}$” OR “PM_1”)

3. Topic = (“nitrogen oxide” OR “nitrogen dioxides” OR “NO_2”)

4. Topic = (“sulfur oxide” OR “sulfur dioxides” OR “SO_2”)

5. Topic = (“ozone” OR “Ground Level Ozone” OR “O_3”)

6. Topic = (“carbon monoxide”)
Outcome:
1. Topic = (“obesit*” OR “obese”)
2. Topic = (“overweight*”)
3. Topic = (“body weight*”)
4. Topic = (“abdominal obesit*” OR “abdominal overweight” OR “central obesit*” OR “visceral obesit*”)
5. Topic = (“body mass index” OR “BMI” OR “Quetelet Index”)
6. Topic = (“waist circumference” OR “WC”)
7. Topic = (“waist-hip ratio” OR “waist to hip ratio” OR “WHR”)
8. Topic = (“fat body” OR “body fat” OR “adipose tissue”)
Object:
Topic = (“child*” OR “adolescen*” OR “juvenile*” OR “youth*” OR “teen*”OR “kid” OR “kids” OR “pubescent*” OR “pubert*” OR “youngster*” OR “minor*” OR “student*” OR “pupil*” OR “preschooler*” OR “schoolchild”)
<u>**Embase:**</u> **2491 records**
Exposure (1 OR 2 OR 3 OR 4 OR 5 OR 6) AND **Outcome** (1 OR 2 OR 3 OR 4 OR 5 OR 6OR 7 OR 8) AND **Object** ()
Exposure:
1. ‘air pollution’/exp OR ‘air pollutant’/exp OR ‘air quality’/exp
2. ‘particulate matter’/exp OR ‘ambient air pollution’ OR ‘ambient particulate matter’ OR ‘fine particulate matter’/exp OR ‘PM_{10}’ OR ‘$PM_{2.5}$’ OR ‘PM_1’
3. ‘nitrogen dioxide’/exp OR ‘NO_2’
4. ‘sulfur dioxide’/exp OR ‘SO_2’
5. ‘ozone’/exp OR ‘Ground Level Ozone’ OR ‘O_3’
6. ‘carbon monoxide’/exp
Outcome:
1. ‘obesity’/exp OR ‘obese’
2. ‘overweight’/exp
3. ‘body weight’/exp
4. ‘abdominal obesity’/exp OR ‘abdominal overweight’ OR ‘central obesity’ OR ‘visceral obesity’
5. ‘body mass index’/exp OR ‘BMI’
6. ‘waist circumference’/exp OR ‘WC’
7. ‘waist hip ratio’/exp OR ‘WHR’
8. ‘body fat’/exp OR ‘fat body’ OR ‘adipose tissue’
Object:
‘child’/exp OR ‘adolescent’/exp OR ‘juvenile’ OR ‘youth’ OR ‘teen’ OR ‘kid’ OR ‘pubescent’ OR ‘puberty’ OR ‘youngster’ OR ‘minor’ OR ‘student’ OR ‘pupil’ OR ‘preschooler’ OR ‘schoolchild’
<u>**Cochran trials:**</u> **69 records**
(“air pollution” OR “air pollutants” OR “air pollut*” OR “air quality” OR “air environmental pollut*” OR “environmental air pollut*” OR “ambient air pollut*” OR “particulate matter” OR “particulate matter” OR “ambient air particulates” OR “airborne particulate matter” OR “particulate air pollutants” OR “ambient particulate matter” OR “fine particles” OR “fine particulate matter” OR “PM_{10}” OR “$PM_{2.5}$” OR “PM_1” OR “nitrogen dioxide” OR “nitrogen dioxide” OR “NO_2” OR “sulfur dioxide” OR “sulfur dioxides” OR “sulfur dioxide” OR “SO_2” OR “ozone” OR “ozone” OR “Ground Level Ozone” OR “O_3” OR “carbon monoxide” OR “carbon monoxide”) in Title Abstract Keyword AND (“obesity” OR “obesit*” OR “obese” OR “overweight” OR “overweight*” OR “body weight” OR “body weight*” OR “obesity, abdominal” OR “abdominal obesit*” OR “abdominal overweight” OR “central obesit*” OR “visceral obesit*” OR “body mass index” OR “body mass index” OR “BMI” OR “Quetelet Index” OR “waist circumference” OR “waist circumference” OR “WC” OR “waist-hip ratio” OR “waist-hip ratio” OR “waist to hip ratio” OR “WHR” OR “fat body” OR “fat body” OR “body fat” OR “adipose tissue” OR “adipose tissue”) in Title Abstract Keyword AND (“child” OR “adolescent” OR “child*” OR “adolescen*” OR “juvenile*” OR “youth*” OR “teen*” OR “kid” OR “kids” OR “pubescent*” OR “pubert*” OR “youngster*” OR “minor*” OR “student*” OR “pupil*” OR “preschooler*” OR “schoolchild”) in Title Abstract Keyword

Supplementary Tables and Figures

Supplementary Table S1. Study quality assessment of the 15 studies that were included in the meta-analysis.

Study ID Criteria	1	2	3	4	5	6	7	8	9	10	11	12	13	14	15
1. Was the research question or objective in this paper clearly stated?	1	1	1	1	1	1	1	1	1	1	1	1	1	1	1
2. Was the study population clearly specified and defined?	1	1	1	1	1	1	1	1	1	1	1	1	1	1	1
3. Was the participation rate of eligible persons at least 50%?	1	1	1	1	1	1	1	1	1	1	1	1	1	1	1
4. Were all the subjects selected or recruited from the same or similar populations(including the same time period)? Were inclusion and exclusion criteria for being in the study pre-specified and applied uniformly to all participants?	1	1	1	0	1	1	1	1	1	1	1	1	1	1	1
5. Was a sample size justification, power description, or variance and effect estimates provided?	0	0	0	0	0	0	0	0	0	0	0	0	0	0	0
6. For the analyses in this paper, were the exposure(s) of interest measured prior to the outcome(s) being measured?	1	1	1	1	0	0	1	1	1	1	1	1	1	1	0
7. Was the timeframe sufficient so that one could reasonably expect to see an association between exposure and outcome if it existed?	1	1	1	1	1	0	1	1	1	1	1	1	1	1	0
8. For exposures that can vary in amount or level, did the study examine different levels of the exposure as related to the outcome (e.g., categories of exposure, or exposure measured as continuous variable)?	1	1	1	0	1	1	1	1	1	1	1	1	1	1	1
9. Were the exposure measures (independent variables) clearly defined, valid, reliable, and implemented consistently across all study participants?	1	1	1	1	1	1	1	1	1	1	1	1	1	1	1
10. Was the exposure(s) assessed more than once over time?	1	1	1	1	1	1	1	1	1	1	1	1	1	1	1
11. Were the outcome measures (dependent variables) clearly defined, valid, reliable, and implemented consistently across all study participants?	1	1	1	1	1	1	1	1	1	1	0	1	1	1	1
12. Were the outcome assessors blinded to the exposure status of participants?	1	1	1	1	1	1	1	1	1	1	1	1	1	1	1
13. Was loss to follow-up after baseline 20% or less?	1	1	1	1	1	1	1	1	0	1	1	1	0	1	1
14. Were key potential confounding variables measured and adjusted statistically for their impact on the relationship between exposure(s) and outcome(s)?	1	1	1	1	1	1	1	1	1	1	1	1	1	1	1
**Sum score**	13	13	13	11	12	11	13	13	12	13	12	13	12	13	11

Supplementary Table S2. The summary results of all meta-analyses of air pollution on childhood obesity and BMI.

Exposure	Eligible Studies	OR/β (95% CI)	*p*-Value	I^2(%)	Q Test (*p*-Value)	*P* of Egger's Test
**Obesity**						
PM_{10}	9	1.12 (1.06, 1.18)	0.000	85.9	56.72 (0.000)	0.076
$PM_{2.5}$	11	1.28 (1.13, 1.45)	0.000	86.3	73.24 (0.000)	0.238

PM_{1}	3	1.41 (1.30, 1.53)	0.000	0	0.20 (0.905)	0.324
O_{3}	2	1.08 (0.99, 1.78)	0.090	71.5	3.51 (0.061)	-
NO_{2}	11	1.11 (1.06, 1.18)	0.000	84.1	62.82 (0.000)	0.001
**BMI**						
PM_{10}	3	0.08 (0.03, 0.12)	0.000	89.1	18.38 (0.000)	0.018
$PM_{2.5}$	3	0.11 (0.05, 0.17)	0.000	82.6	11.49 (0.003)	0.131
NO_{2}	5	0.03 (0.01, 0.04)	0.003	48.6	7.78 (0.100)	0.156
NO_{X}	2	0.18 (−0.09, 0.45)	0.200	91.0	11.07 (0.000)	-

Abbreviations: PM_{10}, particulate matter with the diameter ≤ 10 mm; $PM_{2.5}$, particulate matter with diameter ≤ 2.5 mm; PM_{1}, particulate matter with the diameter ≤ 1 mm; NO_{2}, nitrogen dioxide; NO_{x}, nitrogen oxides; O_{3}, ozone; NRP: near-roadway pollution exposure; BMI, body mass index; OR, odds ratio; β, regression coefficient.

Supplementary Table S3. The summary results of subgroup analysis.

Exposure	Eligible Studies	OR (95% CI)	*p*-Value	I^2 (%)	Q Test (*p*-Value)
**Obesity**					
**Study design (Cohort)**					
PM_{10}	3	1.03 (1.02, 1.05)	<0.001	0.00	0.26 (0.88)
$PM_{2.5}$	2	1.02 (0.33, 1.71)	0.004	0.00	0.14 (0.71)
NO_{2}	4	1.05 (0.97, 1.14)	<0.001	60.57	9.23 (0.26)
**Study design (Cross-sectional)**					
PM_{10}	6	1.08 (1.06, 1.11)	<0.001	87.80	40.97 (<0.00)
$PM_{2.5}$	9	1.26 (1.11, 1.40)	<0.001	86.69	57.37 (<0.00)
NO_{2}	7	1.12 (1.06, 1.19)	<0.001	67.01	18.81 (<0.00)
**Country (China)**					
PM_{10}	5	1.18 (1.05, 1.30)	<0.001	92.65	34.04 (<0.00)
$PM_{2.5}$	5	1.30 (1.15, 1.46)	<0.001	84.54	31.94 (<0.00)
NO_{2}	6	1.15 (1.11, 1.19)	<0.001	0.02	7.29 (0.20)
**Country (Others)**					
PM_{10}	4	1.03 (1.02, 1.05)	<0.001	0.00	2.13 (0.54)
$PM_{2.5}$	6	1.01 (0.94, 1.08)	<0.001	20.71	6.31 (0.28)
NO_{2}	5	1.01 (1.00, 1.02)	<0.001	47.87	7.67 (0.10)
**Study quality (≥13)**					
PM_{10}	5	1.21 (1.09, 1.33)	<0.001	79.40	27.94 (<0.00)
$PM_{2.5}$	7	1.25 (1.10, 1.39)	<0.001	89.55	52.90 (<0.00)
NO_{2}	6	1.13 (1.05, 1.21)	<0.001	75.89	18.08 (<0.00)
**Study quality (<13)**					
PM_{10}	4	1.04 (1.02, 1.05)	<0.001	49.31	4.4 (0.22)
$PM_{2.5}$	4	1.32 (0.77, 1.89)	<0.001	29.83	4.28 (0.23)
NO_{2}	5	1.07 (1.0, 1.15)	<0.001	71.23	16.65 (<0.00)

Abbreviations: PM_{10}, particulate matter with the diameter ≤ 10 mm; $PM_{2.5}$, particulate matter with diameter ≤ 2.5 mm; PM_{1}, particulate matter with the diameter ≤ 1 mm; NO_{2}, nitrogen dioxide; NO_{x}, nitrogen oxides; O_{3}, ozone; NRP: near-roadway pollution exposure; BMI, body mass index; OR, odds ratio; β, regression coefficient.

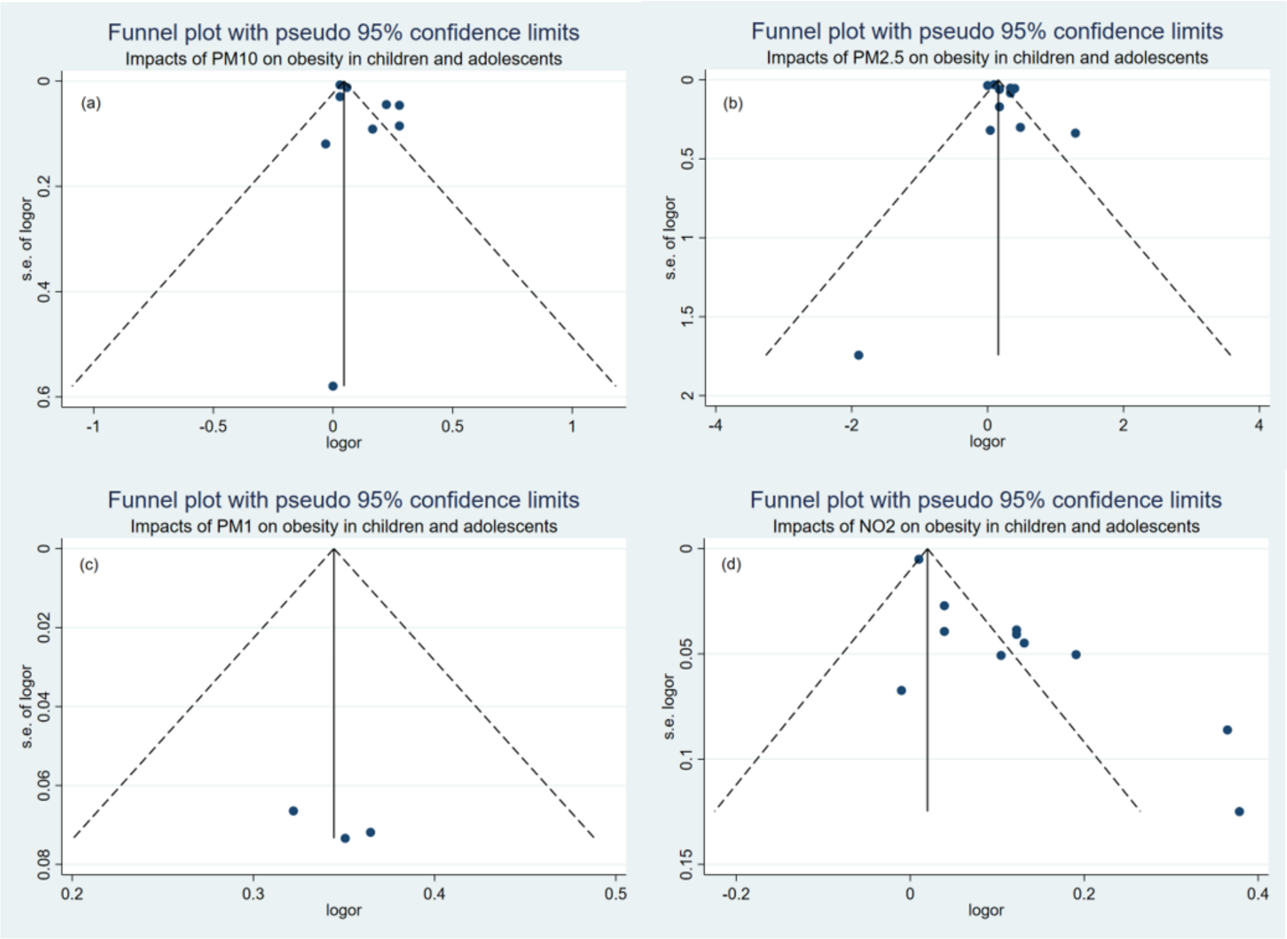

Supplementary Figure S1. Funnel study in the meta-analysis on the association between PM_{10} (**a**), $PM_{2.5}$ (**b**), PM_1 (**c**), and NO_2 (**d**) exposure and childhood obesity.

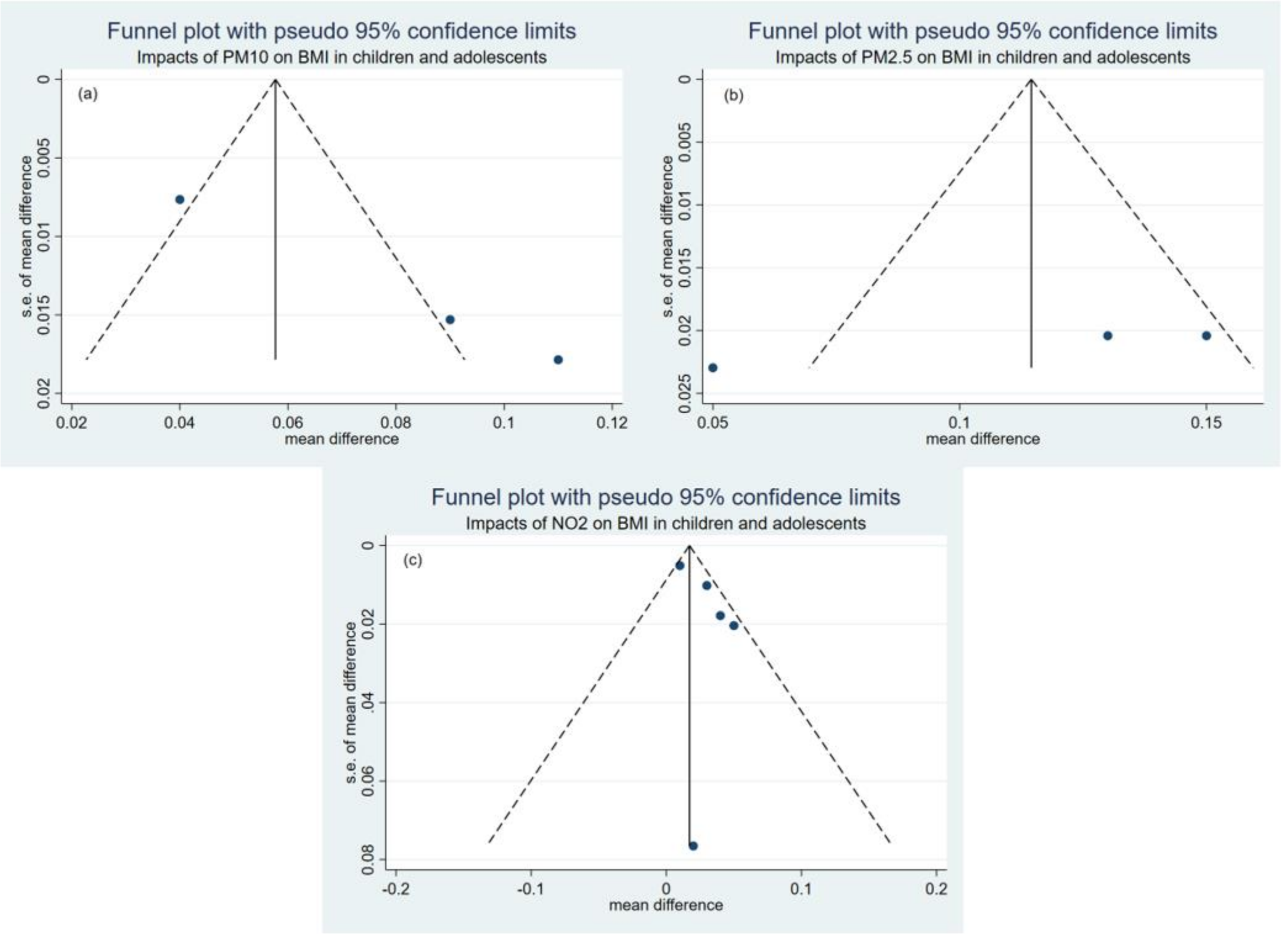

Supplementary Figure S2. Funnel study in the meta-analysis on the association between PM_{10} (**a**), $PM_{2.5}$ (**b**), and NO_2 (**c**) exposure and childhood BMI status.

PM_{10}

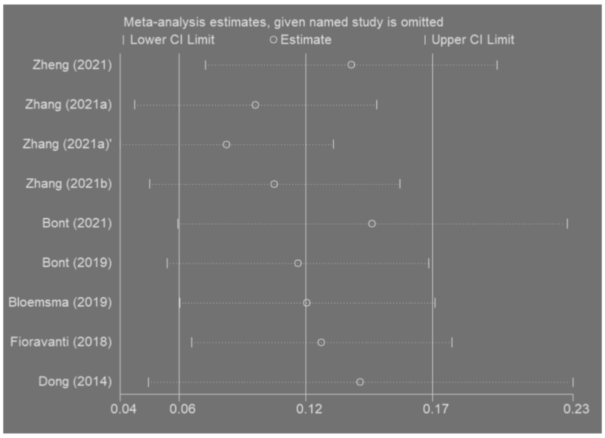

$PM_{2.5}$

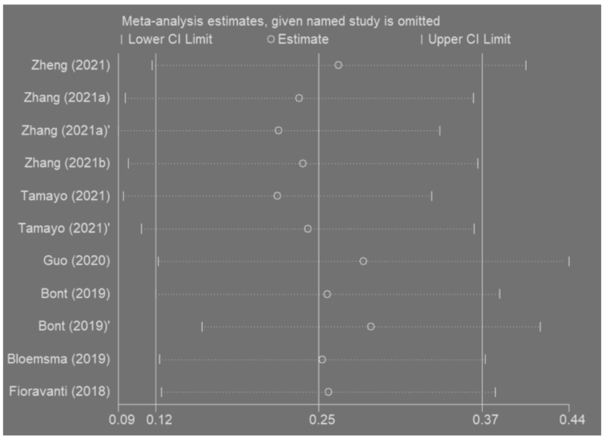

PM_1

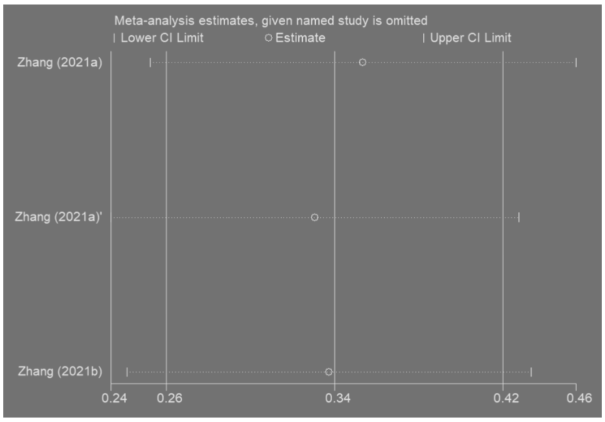

NO_2

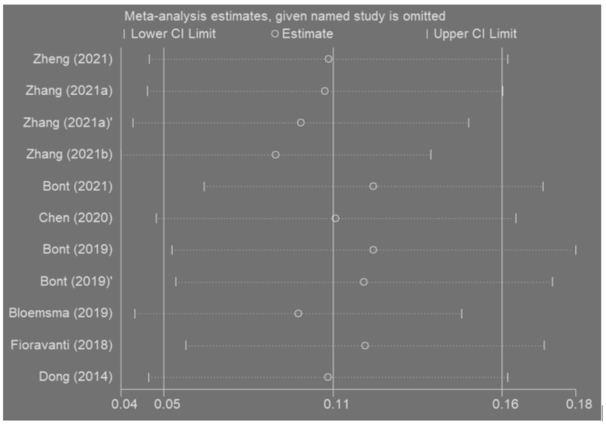

Supplementary Figure S3. Sensitivity analysis of PM_{10}, $PM_{2.5}$, PM_1, and NO_2 on childhood obesity.

PM_{10}

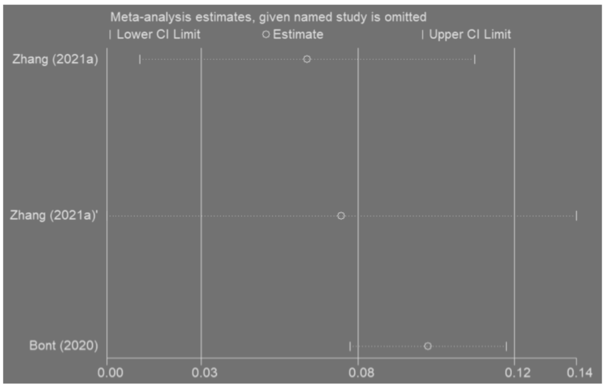

$PM_{2.5}$

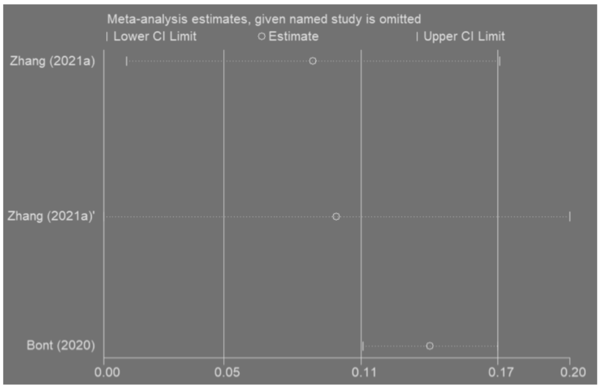

NO_2

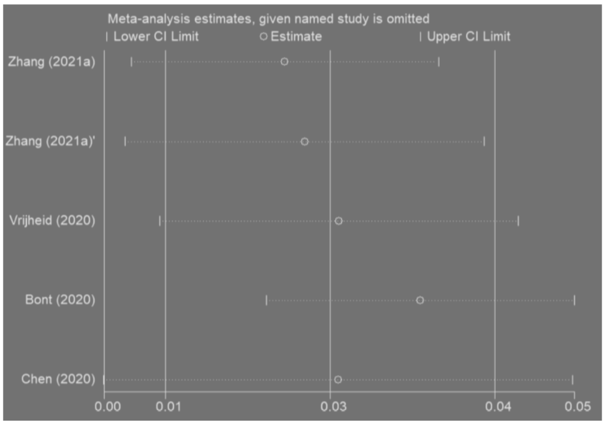

Supplementary Figure S4. Sensitivity analysis of PM_{10}, $PM_{2.5}$, and NO_2 on BMI of children and adolescents.
